# Resting metabolic rate fluctuations across the menstrual cycle: a systematic review

**DOI:** 10.3389/fphys.2026.1778735

**Published:** 2026-03-27

**Authors:** Anežka Hurtová, Marta Gimunová, Michaela Beníčková

**Affiliations:** Department of Physical Activities and Health Sciences, Faculty of Sports Studies, Masaryk University, Brno, Czechia

**Keywords:** basal metabolic rate, energy expenditure, follicular phase, luteal phase, ovarian cycle, resting energy expenditure, resting metabolism

## Abstract

**Introduction:**

The aim of this systematic review was to evaluate the current evidence regarding resting metabolic rate fluctuations across menstrual cycle phases in healthy, naturally menstruating females.

**Methods:**

A systematic search was conducted in the PubMed and Web of Science databases for articles published from January 2020 to September 2025. Of the 331 records identified, seven studies met the eligibility criteria and were included.

**Results:**

Five studies reported a trend toward an elevated metabolic rate during the luteal phase, with four reporting statistically significant increases. The estimated increase ranged from approximately 30 to 120 kcal/day.

**Conclusion:**

However, the magnitude of this physiological shift appears to be small (approximately 3–5%) and may overlap with typical day-to-day biological variability, methodological limitations, or measurement error, potentially contributing to inconsistent findings across studies. Future research should prioritise longitudinal, repeated-measures designs with confirmed ovulation to accurately quantify menstrual cycle-related metabolic fluctuations.

## 1 Introduction

In the fields of nutritional and exercise science, much of the research has historically focused on the male population, resulting in many evidence-based recommendations being primarily grounded in male physiological needs (Wohlgemuth et al., 2021). However, female physiology is inherently specific and is influenced by a range of unique factors, particularly the regular hormonal fluctuations occurring during the menstrual cycle (Sims et al., 2023). While pre-pubescent children display no major sex differences in energy metabolism (Grund et al., 2000), in adulthood females exhibit unique nutritional and metabolic requirements compared to males due to differences in body composition, hormonal profile, and metabolic pathways (Sims et al., 2023). For instance, females typically have a higher percentage of body fat and a lower percentage of muscle mass, which is generally associated with a lower overall resting metabolic rate (RMR) compared to men, even after accounting for body size and composition.

RMR, also referred to as resting energy expenditure, represents the minimum amount of energy required to maintain vital physiological functions (including essential cellular, organ, and neurophysiological functions necessary to maintain homeostasis) at rest and accounts for approximately 60–75% of total daily energy expenditure (Bittencourt et al., 2023; Löfberg et al., 2024). Understanding how the menstrual cycle influences RMR is therefore essential, as it not only expands fundamental physiological knowledge but also has practical implications for applied nutrition strategies, exercise programming, and training load management in physically active females.

The hormonal changes throughout the menstrual cycle, particularly fluctuations in oestrogen and progesterone, play a key role in the regulation of systemic metabolism and substrate utilisation at rest. Oestrogen has been associated with metabolic adaptations that favour lipid oxidation over carbohydrate utilisation, which may influence energy metabolism under resting conditions (Hackney, 2021). In contrast, progesterone is known to exert a thermogenic effect and to modulate glucose metabolism and glycogen storage, particularly during the luteal phase, potentially contributing to an alteration in resting energy expenditure (Smith-Ryan et al., 2022; Wohlgemuth et al., 2021). This interplay of hormones leads to the physiological hypothesis that RMR is not constant across the menstrual cycle. Specifically, the simultaneous peak in oestrogen and progesterone during the mid-luteal phase is hypothesised to increase whole-body energy expenditure, potentially resulting in a higher RMR in the luteal phase compared to the follicular phase (Sims et al., 2023).

Despite well-described physiological mechanisms linking ovarian hormones to energy metabolism, the precise quantitative relationship between menstrual cycle phases and RMR remains incompletely understood. Existing studies report mixed findings and lack consensus. Although previous literature, including a systematic review by Benton et al. (2020), reports an observed increase in RMR during the luteal phase, the overall effect size remains small, and variations in study design contribute to the inconsistencies. Some studies report a statistically significant increase in RMR during the luteal compared to the follicular phase (Malo-Vintimilla et al., 2024; Maury-Sintjago et al., 2022), while others find no significant difference between phases (Gould et al., 2021; Kuikman et al., 2024).

Therefore, a systematic review and analysis of the most recent literature published since the previous review (Benton et al., 2020) is warranted to synthesise current evidence, address methodological heterogeneity, and clarify whether menstrual cycle-related changes in RMR are sufficiently consistent to inform applied nutritional and exercise-related recommendations. The aim of this systematic review is to evaluate and synthesise current evidence on whether and how the menstrual cycle influences RMR in healthy, naturally menstruating females.

## 2 Methods

A systematic review was conducted in accordance with the Preferred Reporting Items for Systematic Reviews and Meta-Analyses (PRISMA) 2020 guidelines (Page et al., 2021). No formal protocol was registered prior to the conduct of this review. However, eligibility criteria and methods were defined *a priori* and strictly followed.

### 2.1 Eligibility criteria

Eligibility criteria were defined *a priori* using the PECO framework: Participants (P): healthy, eumenorrheic females of reproductive age who were not using hormonal contraception. Exposure (E): assessment of at least two menstrual cycle phases. Comparison (C): within-subject comparisons of RMR across different menstrual cycle phases. Outcomes (O): any reported change in RMR (i.e., increase, decrease or no change) between menstrual cycle phases.

Studies were excluded if they involved participants who were pregnant, breastfeeding, or diagnosed with metabolic, neuromuscular, cardiovascular, or psychiatric disorders. Health status was determined based on participants’ self-reported medical history or through structured clinical interviews.

### 2.2 Search strategy and study selection

A search strategy was conducted in two electronic databases – PubMed and Web of Science. The initial search was performed in October 2024 and updated in September 2025. Only articles published from January 2020 to September 2025 were eligible for inclusion. This time restriction was applied to ensure that the present review builds upon, rather than duplicates, the evidence synthesised in the most recent systematic review and meta-analysis on this topic which synthesised literature published prior to 2020 (Benton et al., 2020). As the aim of the current review was to update the evidence base, overlapping searches were not considered necessary.

The following search terms were used: (“menstrual cycle” OR “menstrual cycles” OR “endometrial cycle” OR “endometrial cycles” OR “menstruation” OR “ovarian cycle” OR “ovarian cycles” OR “follicular phase” OR “luteal phase” OR “ovulation”) AND (“basal metabolism” OR “basal metabolic rate” OR “basal metabolic rates” OR “resting metabolic rate” OR “resting metabolic rates” OR “energy metabolism” OR “energy expenditure”).

All retrieved articles were imported into Rayyan software for screening and selection (Ouzzani et al., 2016). Two independent authors (AH, MB) screened all titles, abstracts, and full texts. No discrepancies occurred during the selection process; therefore, involvement of a third reviewer (MG) was not required.

**Figure 1:**
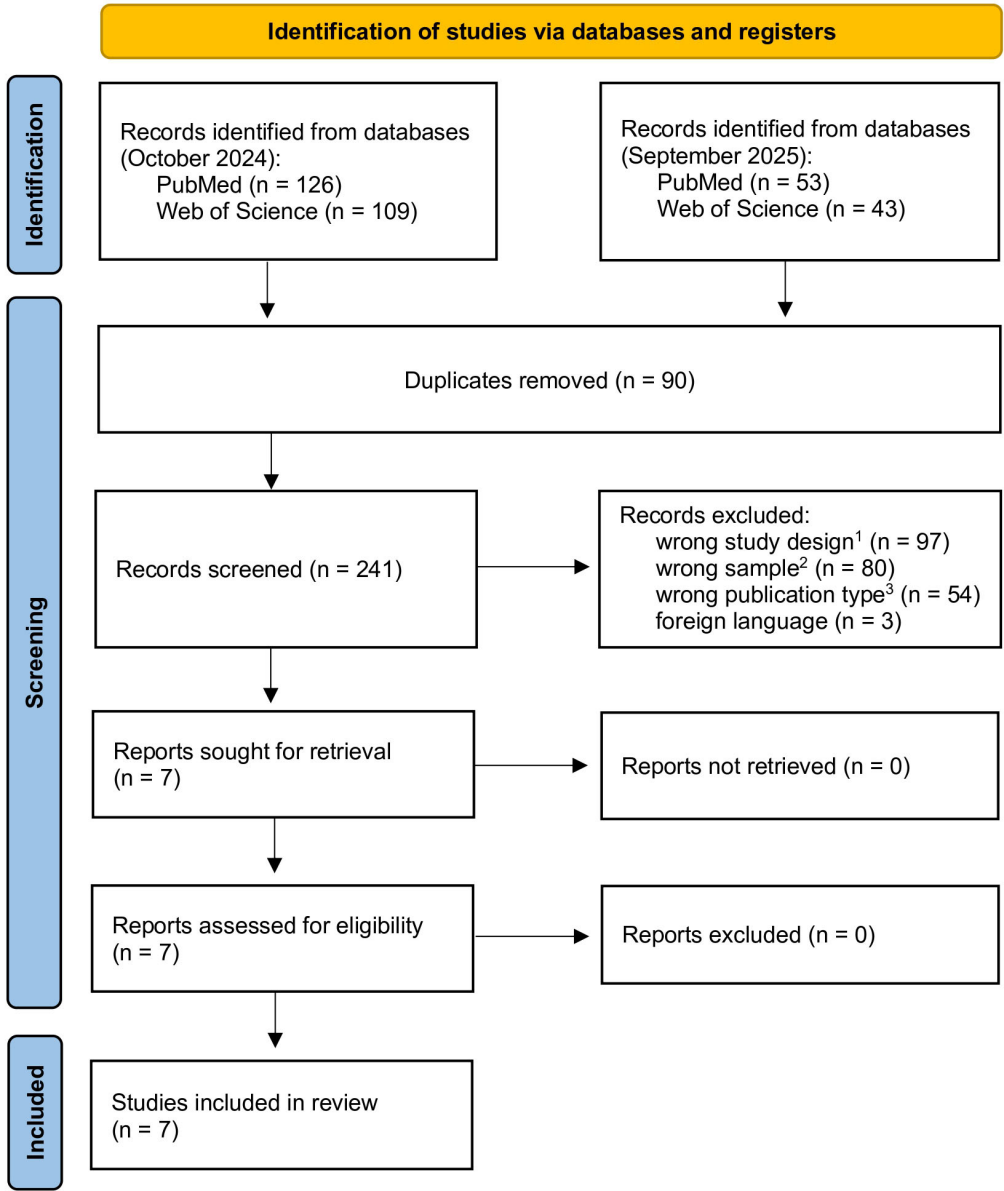
PRISMA flow diagram of the study selection process. ^1^inappropriate study design (e.g., menstrual cycle not considered, or other differing conditions), inappropriate outcomes (e.g., physical or cognitive performance, biochemical or physiological parameters) or both; ^2^ineligible population (e.g., animals, cell models, men, older adults); ^3^publication type not eligible (e.g., book chapters, reviews, conference paper, theses).

Duplicate records were removed prior to screening. Non-English articles, reviews, book chapters, and studies involving animals or cell models were excluded. Studies measuring metabolic rate exclusively during or immediately after exercise were also excluded. The full study selection process is presented in the PRISMA flow diagram (**Figure 1**).

### 2.3 Assessment of study quality

Study quality was assessed using a modified version of the Downs and Black Quality Assessment Checklist (Downs & Black, 1998). A subset of 12 items from the original 27-item tool was retained, covering five domains: reporting quality, external validity, internal validity (bias), internal validity (confounding/selection bias), and statistical power. Each item was scored as “yes” (1 point), “no” or “unable to determine” (0 points). Items rated as “unable to determine” were excluded from the denominator when calculating the percentage score.

All assessments were performed in Microsoft Excel. Because several items were removed from the original checklist, the scoring system was modified, and results are presented as percentages. Following Meignié et al. (2021), studies scoring < 45.4% were classified as having “poor” methodological quality; 45.4–61.0% as “fair” methodological quality; and > 61.0% as “good” methodological quality.

### 2.4 Data collection process

Given that two included studies assessed sleeping metabolic rate (SMR) rather than resting metabolic rate (RMR), and because SMR reflects a closely related physiological construct capturing basal energy expenditure under highly controlled conditions, studies assessing SMR were included in the qualitative synthesis, but their methodological differences were acknowledged (Uchizawa et al., 2025; Zhang et al., 2020).

Data from all included studies were extracted and summarised in **Tables 1**-**4**. Data extraction was performed by one author (AH) and independently double-checked by a second author (MB). **Tables 1**, **2** present study characteristics and methodological details, with studies listed alphabetically by the first author’s last name. Specifically, **Table 1** includes information about study and participant characteristics, **Table 2** summarises menstrual cycle assessment methods, phase descriptions and ovulatory status. **Table 3** summarises methodological aspects relevant to metabolic rate measurement, including time of day, fasting duration, ambient temperature, pre-test rest duration and measurement duration. **Table 4** provides an overview of the primary outcomes, focusing on changes in RMR or SMR across menstrual cycle phases.

**Table 1:** Characteristics of the included studies and participants.

First author, year	Origin	N	Population	Age (years)	Weight (kg)	Height (cm)	BMI (kg/m^2^)	MC length (d)
Gould et al., 2021	USA	19	healthy young females	21.3 ± 3.1	65.0 ± 2.2	166.9 ± 5.7	23.4 ± 1.9	NR
Kuikman et al., 2024	Australia	7	female rugby league players	20.8 ± 3.2	70.8 ± 8.1	NR	27.1 ± 3.4	NR
Löfberg et al., 2024	Finland	38	healthy untrained females	29.8 ± 3.9	68.3 ± 10.9	166.0 ± 6.0	24.9 ± 4.0	27 ± 2
Malo-Vintimilla et al., 2024	Chile	19	healthy females	25.2± 5.1	58.3 ± 7.1	162.0 ± 6.0	22.2 ± 2.2	29 ± 3
Maury-Sintjago et al., 2022	Chile	15	heathy lean females	18–25	56.4 (54.0–57.9)^#^	NR	NR (lean)	NR
Uchizawa et al., 2025	Japan	17 (7 + 10)	7 highly trained runners (R); 10 healthy sedentary females (C)	R: 19.3 ± 0.8 C: 22.6 ± 2.3	R: 49.3 ± 4.7 C: 54.4 ± 8.7	R: 161.2 ± 4.3 C: 162.3 ± 7.2	R: 18.9 ± 1.5 C: 20.6 ± 2.2	25–38
Zhang et al., 2020	Japan	9	healthy females	23.3 ± 1.1	53.9 ± 9.3	161.8 ± 5.5	< 25	NR

Values are expressed as mean ± SD unless otherwise noted. # Median (IQR). BMI, Body Mass Index; C, Controls; IQR, Interquartile Range; MC, Menstrual Cycle; N, Sample size; NR, Not Reported; R, Runners.

**Table 2:** Assessment of menstrual cycle phase and ovulatory status in the included studies.

First author, year	Method of assessment	Phase monitored	Ovulation confirmed	Progesterone concentrations	Note
Gould et al., 2021	self-reported	EFP: d 3.7 ± 1.2 of MC; MFP: 4.7 ± 2.1 d after end of menses	No	NA	–
Kuikman et al., 2024	dual hormone ovulation tests (LH + E); blood samples retrospectively (E + P)	EFP: d 1–5 of MC; LFP: 14–26 h prior to ovulation; MLP: 7 d after ovulation^*^	Yes	Serum P (nmol/l): EFP 1.40 ± 0.60 vs. MLP 43.00 ± 37.90	3 participants excluded due to an insufficient rise in P concentrations during the LP
Löfberg et al., 2024	dual hormone ovulation tests (LH + E); blood samples retrospectively (E + P)	EFP: d 1–5 of MC; MLP: 4–8 d after ovulation^*^	Yes	Serum P (nmol/l): EFP 1.40 ± 0.70 vs. MLP 28.90 ± 8.10	All participants had hormonally indicated ovulations combined with P concentrations over 16 nmol/L during the LP
Malo-Vintimilla et al., 2024	self-reported, blood samples (E + P)	FP: d 5–12 of MC (d 9 ± 2); LP: d 20–25 of MC (d 23 ± 2)	Yes	Serum P (nmol/l^#^): FP 0.95 ± 0.32 vs. LP 20.35 ± 15.58	40% of participants had insufficient rice in P concentration during the LP (lower than 16 nmol/l^#^) FP 0.64 ± 0.32 nmol/l^#^ vs. LP 5.09 ± 3.82 nmol/l^#^
Maury-Sintjago et al., 2022	medical record	FP: d 6–13 of MC; LP: d 15–28 d of MC	No	NA	–
Uchizawa et al., 2025	self-reported	LFP: d 9–13 of MC (d 10.6 ± 1.0) LP: d 21–24 of MC (d 22.4 ± 0.9)	No	NA	–
Zhang et al., 2020	self-reported, urine samples respectively (E + P)	FP: d 9–13 of MC (d 10.4 ± 1.2) LP: d 21–24 of MC (d 22.7 ± 0.9)	NR	Urinary P excretion (ng/10 h): FP 1.28 ± 0.34 vs. LP 2.13 ± 0.93	No explicit note related to ovulatory status

Values are expressed as mean ± SD unless otherwise noted. #Values were converted from ng/ml to nmol/l (1 ng/ml = 3.18 nmol/l); *after a positive ovulation test. d, day; E, Oestrogen; EFP, Early Follicular Phase; FP, Follicular Phase; LFP, Late Follicular Phase; LH, Luteinizing Hormone; LP, Luteal Phase; MC, Menstrual Cycle; MFP, Mid-Follicular Phase; MLP, Mid-Luteal Phase; NR, Not Reported; P, Progesterone; NA, Not Applicable.

**Table 3:** Characteristics of the testing conditions and measurement methods in the included studies.

First author, year	Conditions					Measured variable	Method of measurement
	Start of test	Fasting	Temperature	Pre-test rest	Duration		
Gould et al., 2021	NR	12 h	NR	NR	30 min (first 5 min excluded)	RMR (kcal/d)	Indirect calorimetry (canopy system)
Kuikman et al., 2024	morning	overnight	controlled (NR)	10 min rest +15 min familiarisation	20 min (2 × 10 min collections)	RMR (kcal/d)	Indirect calorimetry (Douglas bags)
Löfberg et al., 2024	morning	10 h	19.5–24.0 °C	10 min	20 min (first 5 min excluded)	REE (kcal/d)	Indirect calorimetry (canopy system)
Malo-Vintimilla et al., 2024	8:30^*^	12 h	thermoneutral (NR)	30 min	20 min	RMR (kJ/d)	Indirect calorimetry (canopy system)
Maury-Sintjago et al., 2022	morning	10–12 h	20–24 °C	30 min	25 min (first 5 min excluded)	RMR (kcal/d)	Indirect calorimetry (canopy system)
Uchizawa et al., 2025	RMR: morning (7:00–8:00) SMR: 23:00–00:00	RMR: ≥ 13.5 h SMR: > 5 h	25 °C	RMR: 8 h sleep (measured in bed) SMR: NR	RMR: 30 min (first & last 5 min excluded) SMR: 8 h (during sleep, mean of lowest 3 consecutive h)	RMR, SMR (kJ/d)	Whole-room indirect calorimeter
Zhang et al., 2020	23:00–24:00	5 h	25.0 ± 0.5 °C	NR	8 h (during sleep)	SMR (kcal/min)	Whole-room indirect calorimeter

*The 8:30 time is derived from the arrival time (8:00) and the duration of the mandatory rest period (30 min). h, hours; NR, Not Reported; REE, Resting Energy Expenditure; RMR, Resting Metabolic Rate; SMR, Sleeping Metabolic Rate.

**Table 4:** Summary of the changes in resting or sleeping metabolic rate across the menstrual cycle.

First author, year	Phases compared	Phase 1 value	Phase 2 value	Mean difference (phase 2 vs. phase 1)	P-value	Effect size
Gould et al., 2021	EFP vs. MFP	1592 ± 234	1598 ± 147	+ 6 kcal/d	0.894	NR
Kuikman et al., 2024	EFP vs. MLP	NR	NR	NR	0.875 (absolute); 0.958 (relative)	NR
Löfberg et al., 2024	EFP vs. MLP	1415 ± 201	1455 ± 194	+ 40 kcal/d	0.061	Cohen’s d = 0.20
Malo-Vintimilla et al., 2024	FP vs. LP	O + AO: 1205 ± 110^#^ O: NR AO: NR	O + AO: 1242 ± 117^#^ O: NR AO: NR	O + AO: + 37 kcal/d^#^ O: + 58 kcal/d^#^ AO: + 8 kcal/d^#^	O + AO: 0.040* O: 0.070 AO: 0.990	O + AO: Cohen’s d = 0.33 O: Cohen’s d = 0.79 AO: Cohen’s d = 0.05
Maury-Sintjago et al., 2022	FP vs. LP	987–1095^†^	1102–1319^†^	+ 121.6 kcal/d	0.004*	NR
Uchizawa et al., 2025	LFP vs. LP	RMR: R: 1199 ± 130^#^ C: 1237 ± 160^#^	RMR: R: 1221 ± 83^#^ C: 1283 ± 183^#^	RMR R: + 22 kcal/d^#^ C: + 46 kcal/d^#^	RMR: >0.050	NR
		SMR: R: 1088 ± 102^#^ C: 1070 ± 174^#^	SMR: R: 1117 ± 108^#^ C: 1125 ± 151^#^	SMR: R: +29 kcal/d^#^ C: +55 kcal/d^#^	SMR: R: 0.035* C: >0.050	NR
Zhang et al., 2020	FP vs. LP	NR	NR	+27 kcal/8h	0.033*	NR

*Significant at *p* < 0.050; # Values were converted from kJ to kcal (1 kcal = 4.184 kJ); ^†^Data presented as interquartile range (P25–P75). AO, Anovulatory Group; C, Controls; d, day; EFP, Early Follicular Phase; FP, Follicular Phase; LFP, Late Follicular Phase; LP, Luteal Phase; MC, Menstrual Cycle; MFP, Mid-Follicular phase; MLP, Mid-Luteal Phase; NR, Not Reported; O, Ovulatory Group; R, Runners; RMR, Resting Metabolic Rate; SMR, Sleeping Metabolic Rate.

## 3 Results

### 3.1 Study selection

A total of 331 records were identified through database searching (PubMed and Web of Science). After the removal of duplicates and screening based on predefined eligibility criteria, seven studies met the inclusion criteria and were included in the final analysis. The selection process is summarised in the PRISMA flow diagram (**Figure 1**).

### 3.2 Methodological quality of included studies

All seven studies scored above 66.7% on the modified Downs and Black checklist and were therefore classified as having good methodological quality. One study achieved the highest possible score (Malo-Vintimilla et al., 2024). Two studies scored 90.0% (Löfberg et al., 2024; Uchizawa et al., 2025), two scored 77.8% (Kuikman et al., 2024; Zhang et al., 2020), one scored 70.0% (Gould et al., 2021), and one scored 66.7% (Maury-Sintjago et al., 2022).

Common methodological limitations included insufficient reporting on population representativeness, limited detail in the presentation of results, and inadequate methods for menstrual cycle verification. Items 11 and 12, which relate to population representativeness, were marked as “unable to determine” (UD) for all studies, as participant recruitment relied on convenience sampling (e.g., posters, advertisements).

### 3.3 Study characteristics

Sample sizes ranged from 7 to 38, with a total of 124 participants across all included studies. All participants were healthy females of reproductive age (18–34 years), were not using hormonal contraception and reported regular menstrual cycles. Mean cycle lengths of 27 ± 2 days and 29 ± 3 days were reported by Löfberg et al. (2024) and Malo-Vintimilla et al. (2024), respectively, while Uchizawa et al. (2025) reported a range of 25–38 days. No data on menstrual cycle length were provided in the remaining four studies. For more details regarding the characteristics of the included studies and participants, see **Table 1**.

Methods used to determine menstrual cycle phase varied considerably (see **Table 2**). Two studies relied solely on self-reported menstrual tracking (Gould et al., 2021; Uchizawa et al., 2025), whereas others employed more rigorous verification methods, including: (i) dual-hormone ovulation tests (LH and oestrogen) (Kuikman et al., 2024; Löfberg et al., 2024); and (ii) serum or urine hormone analysis (Kuikman et al., 2024; Löfberg et al., 2024; Malo-Vintimilla et al., 2024; Zhang et al., 2020). In one study, menstrual cycle information was verified through medical records (Maury-Sintjago et al., 2022).

Explicit confirmation of ovulation using defined progesterone thresholds or ovulation testing was not achieved in all studies. Three studies biochemically verified ovulatory status and reported exclusions or sub-analyses based on these data. In Löfberg et al. (2024), ovulation was confirmed in all participants. On the other hand, Kuikman et al. (2024) excluded three participants from the study due to low progesterone concentrations during the luteal phase, and Malo-Vintimilla et al. (2024) identified that eight of twenty participants had low progesterone concentrations during the luteal phase, classifying these cases as anovulatory in their statistical analysis. While Zhang et al. (2020) confirmed a significant rise in urinary progesterone concentration during the luteal phase, they did not explicitly apply specific thresholds to verify ovulation (see details in **Table 2**).

Timing of RMR or SMR assessments across the menstrual cycle also differed and is detailed in **Table 2**. All included studies measured RMR or SMR across at least two menstrual cycle phases, such as early follicular phase and mid-follicular phase (Gould et al., 2021), early follicular phase and mid-luteal phase (Kuikman et al., 2024; Löfberg et al., 2024), late follicular phase and luteal phase (Uchizawa et al., 2025), or compared follicular and luteal phases (Malo-Vintimilla et al., 2024; Maury-Sintjago et al., 2022; Zhang et al., 2020).

Regarding RMR or SMR measurement methods (**Table 3**), four studies used indirect calorimetry with a canopy system (Gould et al., 2021; Löfberg et al., 2024; Malo-Vintimilla et al., 2024; Maury-Sintjago et al., 2022), Uchizawa et al. (2025) and Zhang et al. (2020) used a whole-room indirect calorimeter, while Kuikman et al. (2024) used indirect calorimetry as well but with Douglas bags.

Measurement conditions were generally comparable across studies. Fasting duration was either overnight (Kuikman et al., 2024) or between 10 and 13.5 hours in most studies, except for Uchizawa et al. (2025) and Zhang et al. (2020), where participants fasted for at least 5 hours. Ambient room temperature ranged from 19.5 to 25 °C. Measurement duration typically ranged from 20 to 30 minutes, except in two studies that assessed SMR, which involved continuous overnight measurement lasting approximately 8 hours (Uchizawa et al., 2025; Zhang et al., 2020).

### 3.4 Changes in resting metabolic rate during the menstrual cycle

**Table 4** provides an overview of changes in RMR or SMR across menstrual cycle phases as reported in the included studies. Of the seven included studies, four reported statistically significant differences in RMR or SMR across the menstrual cycle. Specifically, Malo-Vintimilla et al. (2024) and Maury-Sintjago et al. (2022) observed significantly higher RMR during the luteal phase compared to the follicular phase, with increases ranging from 37 to 122 kcal/d (p = 0.040 and p = 0.004, respectively). Moreover, Malo-Vintimilla et al. (2024) conducted a sub-analysis stratified by ovulatory status. In the ovulatory group (n = 12), RMR increased by 58 kcal/d during the luteal phase, representing a moderate-to-large effect size (Cohen’s d = 0.79), although the result did not reach statistical significance (p = 0.070). In contrast, in the anovulatory group (n = 8), RMR increased by only 8 kcal/d during the luteal phase, corresponding to a trivial effect size (Cohen’s d = 0.05), with no evidence of a phase effect (p = 0.990).

Zhang et al. (2020) reported a significant increase in SMR during the luteal phase compared to the follicular phase (+27 kcal over 8 hours, p = 0.033). Similarly, Uchizawa et al. (2025) found a significantly higher SMR in the luteal phase than in the late follicular phase, but this effect was present only in female runners (+29 kcal/day, p = 0.035); no significant changes were observed across phases in the sedentary subgroup. Löfberg et al. (2024) reported higher resting energy expenditure in the mid-luteal phase compared to the early follicular phase; however, this difference did not reach statistical significance and had only a small effect, although it approached significance (p = 0.061, Cohen’s d = 0.20). The remaining studies did not detect statistically significant differences in RMR across menstrual cycle phases (Gould et al., 2021; Kuikman et al., 2024; Uchizawa et al., 2025).

## 4 Discussion

The aim of this systematic review was to determine whether and how RMR changes across different phases of the menstrual cycle in healthy, naturally menstruating females. Of the six studies that directly compared follicular and luteal phases, five reported an elevation in metabolic rate during the luteal phase. While statistical significance was achieved in four studies (Malo-Vintimilla et al., 2024; Maury-Sintjago et al., 2022; Uchizawa et al., 2025; Zhang et al., 2020), the lack of statistical significance in others (e.g., Kuikman et al., 2024) does not necessarily imply the absence of a physiological effect.

**Figure 2:**
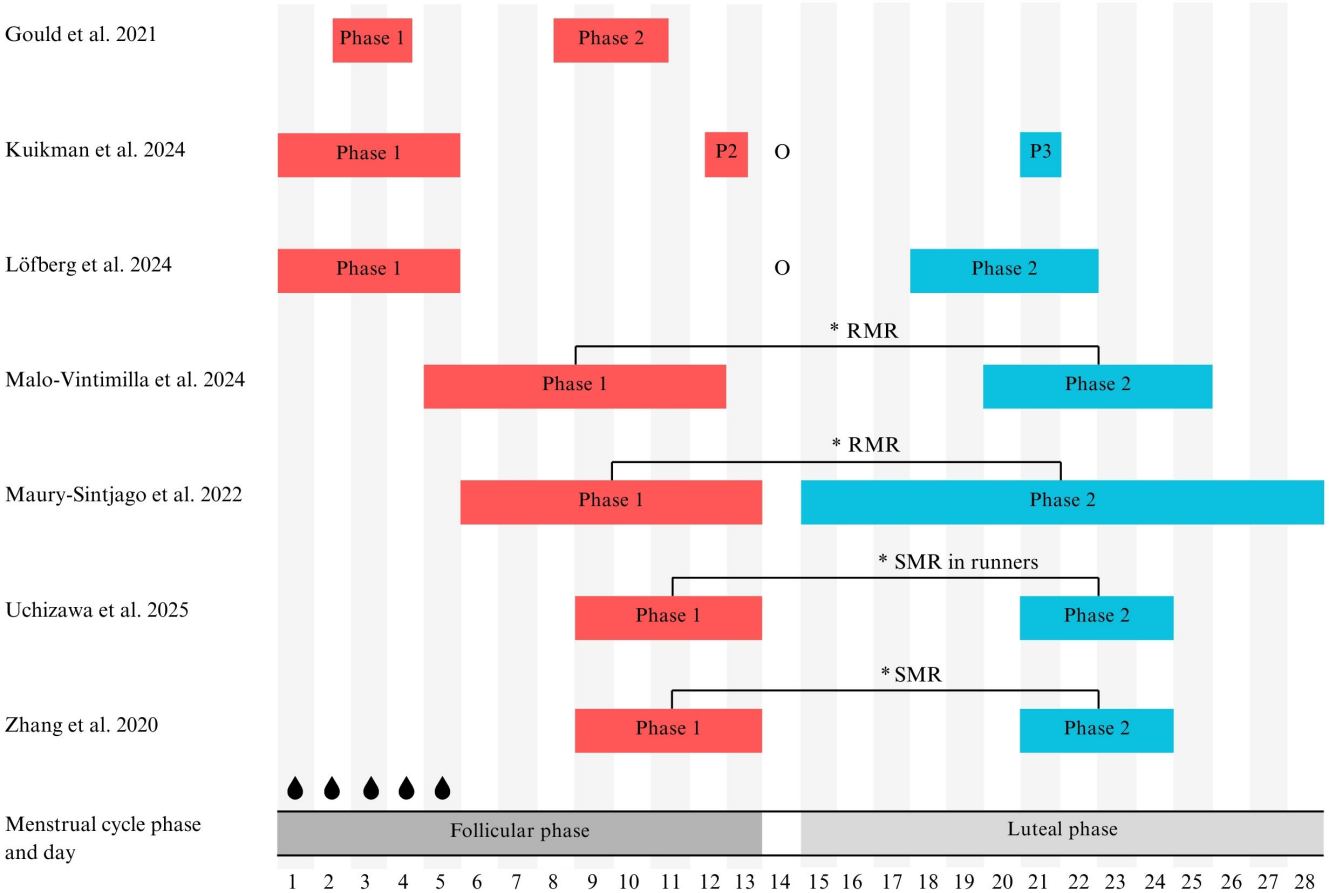
Summary of resting metabolic rate measurement timing and reported significant differences across menstrual cycle phases. * Significant at p < 0.050. O, positive ovulation test (ovulation); P, Phase; RMR, Resting Metabolic Rate; SMR, Sleeping Metabolic Rate.

An important factor in interpreting these mixed findings may lie in the specific timing of measurement across the menstrual cycle phases. As illustrated in **Figure 2**, substantial heterogeneity exists in the exact cycle days assessed within both the follicular and luteal phases across studies. Notably, Gould et al. (2021) observed stable RMR between the early and mid-follicular phases, suggesting that metabolic rate remains relatively stable throughout the follicular phase despite rising oestrogen levels. On the other hand, Malo-Vintimilla et al. (2024) found that RMR was significantly lower in the follicular phase compared to the luteal phase. However, when considering ovulation status, the difference was more pronounced in females who ovulated (ΔRMR: 58 ± 39 kcal/d), while it was nearly absent in anovulatory females (ΔRMR: 8 ± 102 kcal/d). This suggests that progesterone-driven metabolic changes during the luteal phase may be more relevant in ovulatory cycles, reinforcing the importance of distinguishing between ovulatory and anovulatory cycles in research on menstrual cycle effects on RMR. Consequently, variations in measurement timing within the follicular phase (e.g., day 2 vs. day 10) may contribute to some variability; however, accurately capturing the mid-luteal progesterone peak may be of greater physiological relevance. Importantly, the lack of statistical significance in some studies comparing follicular and luteal phases likely reflects the difficulty of detecting a relatively small metabolic shift against a background of high inter-individual variability and methodological noise. Although most studies reported a luteal phase increase in RMR, the estimated effect size is small and heavily overlaps with the typical measurement error and day-to-day biological variability, which typically ranges from 3% to 5% (Compher et al., 2006).

Indeed, our findings align with previous literature suggesting a small but potentially measurable metabolic shift favouring the luteal phase. A systematic review and meta-analysis by Benton et al. (2020) reported a small but significant overall effect favouring increased RMR during the luteal phase (p < 0.001). However, Benton et al. (2020) also highlighted that in studies published after the year 2000, which typically employ stricter methodologies, the overall effect size was substantially reduced and often failed to reach statistical significance. Our review reflects this complexity. While we identified high-quality studies that found significant increases in RMR (Malo-Vintimilla et al., 2024) or SMR (Uchizawa et al., 2025) during the luteal phase, the lack of significance in the remaining studies supports the notion that the physiological variability inherent to the menstrual cycle is subtle and may often fall within the typical day-to-day measurement error of RMR assessments.

The inconsistency in findings across the literature should be interpreted in the context of measurement variability. The majority of included studies met the best practice considerations for RMR measurement established by Compher et al. (2006), ensuring a baseline of methodological rigor. However, some reporting gaps remained; specifically Gould et al. (2021) did not fully detail the start time of measurement, ambient temperature, or pre-test rest duration, while Malo-Vintimilla et al. (2024) and Kuikman et al. (2024) did not specify whether the initial minutes of RMR measurement were excluded to ensure steady state. Even when these methodological standards are met, the interpretation of RMR changes is complicated by the overlap between the magnitude of the physiological effect and measurement error. The expected error of measurement, as well as day-to-day biological variability, typically ranges from 3% to 5% (Compher et al., 2006). Since the estimated increase in RMR due to the menstrual cycle is often small, it can be easily obscured if the measurement error is not minimised (Benton et al., 2020; Henry et al., 2003).

This issue is further compounded by intra-individual variability, as highlighted by Henry et al. (2003). Their research demonstrated that females do not respond uniformly. They identified distinct metabolic phenotypes, distinguishing between a “low variability” (coefficient of variation 2–4%) group comparable to males, and a “high variability” (coefficient of variation 5–10%) group. This implies that RMR should not be assumed to be stable in all females. In studies with small sample sizes, the proportion of “high variability” versus “low variability” participants could significantly skew the results. If a cohort predominantly consists of metabolically stable females, cycle-related changes may appear negligible. Conversely, in females with high intrinsic variability, a single measurement in each phase may fail to capture the true mean RMR. Thus, the “null” results reported by Kuikman et al. (2024) and in the sedentary controls of Uchizawa et al. (2025) may not indicate a lack of physiological effect, but rather the masking of individual responses by group-level averaging and biological noise.

Taken together, although the available evidence suggests that metabolic rate is higher in the luteal phase compared to the follicular phase, inconsistencies across studies prevent definitive conclusions. The findings of this review are largely in agreement with those of Benton et al. (2020), with both highlighting the need for improved study methodologies to establish a clearer understanding of menstrual cycle-related metabolic changes. Importantly, although several studies report a luteal phase increase in RMR, the magnitude of this effect appears to be small and may overlap with typical measurement error (3–5%), which could partly explain the inconsistent findings across studies. Moreover, the practical significance of these observed changes (30–120 kcal/day) may have limited practical implications for most individuals in the general population. However, these fluctuations could be highly relevant in applied sports nutrition, particularly for elite athletes or individuals at risk of low energy availability.

### 4.1 Physiological mechanisms

The observed metabolic shift toward increased energy expenditure in the luteal phase has traditionally been attributed to the thermogenic effect of progesterone, which centrally elevates the thermoregulatory set-point and core body temperature (Nakayama et al., 1975). This increase is typically measured at approximately 0.20–0.30 °C during ovulatory menstrual cycles (Hackney and Constantini, 2020). While the rise in core body temperature during the luteal phase is often cited as the main reason for increased RMR, recent data suggest this thermal effect alone cannot explain the magnitude of the metabolic increase. For example, Zhang et al. (2020) reported a 6.9% increase in SMR alongside a minor 0.27 °C rise in core body temperature. Physiologically, such a small increase in body temperature would typically result in only a slight metabolic rise. However, the observed increase was large, substantially greater than what would be expected from the temperature shift alone. This suggests that the rise in energy expenditure may not be solely a byproduct of increased body temperature and could involve direct metabolic effects of sex hormones or other non-thermal mechanisms (Zhang et al., 2020).

One of the key non-thermal mechanisms proposed to explain this discrepancy is the involvement of the sympathetic nervous system. Research confirms that the mid-luteal phase is associated with significantly elevated muscle sympathetic nerve activity compared to the early follicular phase (Minson et al., 2000). Progesterone may act synergistically with oestrogen to modulate catecholamine activity and enhance β-adrenergic responsiveness. This heightened sympathetic tone not only supports thermogenesis but may also stimulate metabolic processes that have been proposed to contribute to the elevated resting oxygen consumption observed in the luteal phase independently of temperature changes (Carter et al., 2013).

Furthermore, the interplay between oestrogen and progesterone appears to modulate substrate utilisation, independently of total energy expenditure. Oestrogen has been associated with increased lipolysis and lipid oxidation, alongside a relative sparing of muscle glycogen during submaximal exercise intensities (Oosthuyse et al., 2023). This physiological tendency may be reflected in the respiratory quotient, which serves as an indirect marker of substrate oxidation. For instance, Malo-Vintimilla et al. (2024) found marginally higher respiratory quotient values in the follicular phase (0.84) compared to the luteal phase (0.81), which may suggest a modest shift toward greater reliance on lipid substrates during the luteal phase, although such differences should be interpreted cautiously given the indirect nature of this marker. However, this relationship is complex due to the concurrent effects of progesterone. While oestrogen has been linked to glycogen-sparing mechanisms, the peaking concentrations of progesterone during the mid-luteal phase may be associated with increased whole-body protein turnover and amino acid oxidation (e.g., phenylalanine, lysine, and leucine) under certain metabolic conditions (Oosthuyse et al., 2023). Consequently, the metabolic phenotype during the luteal phase likely represents a dynamic balance between progesterone-related increases in overall energy expenditure and whole-body protein turnover, alongside a relative oestrogen-mediated reliance on lipid substrates.

### 4.2 Limiting factors

While this systematic review provides valuable insights into the potential effects of the menstrual cycle on RMR, several limitations must be acknowledged. Firstly, there is significant heterogeneity in menstrual cycle verification methods. Several included studies relied solely on self-reported cycle tracking rather than direct hormonal measurements or ovulation tests. Without hormonal confirmation, it is impossible to verify the occurrence of ovulation. As demonstrated by Malo-Vintimilla et al. (2024), the metabolic increase in the luteal phase appears to be associated with progesterone concentrations and is absent in anovulatory cycles. Therefore, studies relying solely on self-reported tracking carry a high risk of phase misclassification. The lack of biochemical confirmation of ovulation likely attenuates true luteal phase effects and may partly explain the null findings observed in some studies. Another limitation involves the heterogeneity in the timing of luteal phase measurements, which was often based on broad calendar-based windows rather than specific ovulatory events. To address this, future studies should verify ovulation using defined progesterone thresholds or dual-hormone testing. Furthermore, researchers should consider excluding anovulatory cycles or conducting stratified analyses, and should standardise luteal measurements relative to ovulation rather than simply relying on calendar days.

Secondly, the interpretation of results is constrained by relatively small sample sizes. Most of the included studies were small and likely underpowered to detect small-to-moderate effects. Moreover, only two included studies performed an *a priori* sample size calculation (Malo-Vintimilla et al., 2024; Uchizawa et al., 2025). Non-significant findings may therefore reflect a type II error rather than a true absence of physiological variation (Sullivan and Feinn, 2021). In the context of high biological variability, as highlighted by Henry et al. (2003), intra-individual variability in RMR can range from 2% to 10%. Given that the estimated thermogenic effect of the menstrual cycle is relatively small, it can be easily masked by this “biological noise” in underpowered studies. This risk of bias is particularly relevant for studies with small cohorts, such as Kuikman et al. (2024). To reduce the risk of type II error and biological noise, future studies should conduct *a priori* power analyses and implement repeated measurements within each menstrual cycle phase.

Thirdly, the majority of the included studies were cross-sectional or short-term, preventing a comprehensive understanding of how RMR fluctuates across multiple consecutive cycles within the same individual. Future research should prioritise longitudinal designs with rigorous hormonal verification (following the best-practice guidelines by Elliott-Sale et al. (2021)) to better isolate the effect of ovarian hormones from measurement error and biological variability.

Fourthly, the inclusion of both RMR and SMR should be considered a methodological limitation. Techniques varied between standard RMR measurements and SMR assessments. Although closely related, differences in construct and measurement duration may affect comparability. Studies utilising SMR (Uchizawa et al., 2025; Zhang et al., 2020) appeared to detect metabolic fluctuations more consistently than those relying on awake RMR measurements. Notably, Uchizawa et al. (2025) measured both parameters in the same cohort of runners and found a significant luteal phase increase in SMR, whereas morning RMR showed no statistical difference. A potential explanation lies in the measurement conditions; SMR protocols minimise variability attributable to skeletal muscle tone, postural maintenance, and psychological arousal, thereby providing a stable physiological baseline. By reducing this “non-hormonal noise”, SMR assessments may allow subtle endocrine-driven thermogenic effects, such as the progesterone-mediated increase in core temperature and energy expenditure, to become statistically detectable, whereas they remain obscured by higher variability in standard RMR protocols (Uchizawa et al., 2025; Zhang et al., 2020). However, current evidence is insufficient to recommend SMR as a superior approach. Future research should clearly distinguish these outcomes and avoid treating them as interchangeable without appropriate justification. Additionally, external influences such as diet and energy availability were inconsistently accounted for. In athletic populations, undetected low energy availability could suppress metabolic rate (Ihalainen et al., 2024), potentially negating the thermogenic effect of progesterone (Kuikman et al., 2024; Uchizawa et al., 2025).

Furthermore, some of the studies did not meet the best practice recommendations for metabolic rate measurement as outlined by Compher et al. (2006), or did not specify methodological details regarding RMR measurements. Variations in fasting duration, time of day, ambient temperature, and the specific calorimetry systems used may influence metabolic rate, reduce comparability, and increase methodological noise. Future studies should strictly adopt standardised pre-test conditions outlined in, for example, Compher et al. (2006), and transparently report methodological details.

Finally, additional limitations of this review should be acknowledged. The review was restricted to studies published in English, which may have led to the exclusion of relevant studies published in other languages. In addition, the number of eligible studies was limited, and there was substantial methodological heterogeneity across the included studies, which should be considered when interpreting the generalisability of the findings. Due to this heterogeneity, a quantitative meta-analysis was not considered appropriate.

## 5 Conclusion

The findings of this systematic review suggest a probable but small luteal phase increase in RMR that is highly sensitive to methodological precision and ovulatory status, rather than representing a definitively consistent trend. Although statistical significance was not achieved in all included studies, five of the six investigations comparing follicular and luteal phases (excluding one study that compared early vs. mid-follicular phases) reported a metabolic increase, typically ranging from 30 to 120 kcal/day (approximately 3–5%). However, current evidence indicates that this effect is subtle and easily obscured by methodological heterogeneity, biological variability, and measurement error. Key factors influencing the detection of these metabolic changes include ovulatory status and measurement sensitivity. As highlighted in this review, metabolic increases are likely restricted to confirmed ovulatory cycles and may be absent in females with an anovulatory menstrual cycle. Furthermore, assessing SMR appears to be more sensitive, although not necessarily superior to standard RMR measurements in detecting these fluctuations, particularly in active populations where energy turnover is higher, likely by minimising external confounders. Importantly, the practical relevance of this subtle metabolic increase is limited for most individuals in the general population, but may increase with sport level (e.g., elite athletes or individuals at risk of low energy availability). Future research should conduct *a priori* sample size calculations and prioritise longitudinal, repeated-measures designs with rigorous hormonal verification of ovulation to ensure the presence of a functional luteal phase. Furthermore, adopting standardised conditions for RMR measurement and implementing more sensitive protocols, such as SMR, could help minimise external variability and better isolate the subtle metabolic effects of the menstrual cycle.

## Data Availability

All relevant data supporting the conclusions of this review are included within the article. Further inquiries can be directed to the corresponding author.
