# Correction: Resting metabolic rate fluctuations across the menstrual cycle: a systematic review

**DOI:** 10.3389/fphys.2026.1854612

**Published:** 2026-04-20

**Authors:** Anežka Hurtová, Marta Gimunová, Michaela Beníčková

**Affiliations:** Department of Physical Activities and Health Sciences, Faculty of Sports Studies, Masaryk University, Brno, Czechia

**Keywords:** Basal metabolic rate, energy expenditure, follicular phase, luteal phase, ovarian cycle, resting energy expenditure, resting metabolism

In the published article, there was a mistake in the **Funding** statement. An incorrect number was provided for the Ministry of Education, Youth and Sports of the Czech Republic (number MUNI/A/1455/2024). The correct number is “MUNI/A/1475/2024”.

The original article has been updated.

